# Telocytes in gastric lamina propria of the Chinese giant salamander, *Andrias davidianus*

**DOI:** 10.1038/srep33554

**Published:** 2016-09-15

**Authors:** Hui Zhang, Shengwei Zhong, Pengcheng Yu, Tingting Ge, Shasha Peng, Xiaoquan Guo, Zuohong Zhou

**Affiliations:** 1 College of Animal Science and Technology, Jiangxi Agricultural University, Nanchang, China

## Abstract

In this study, we attempt to identify gastric telocytes (TCs) of the Chinese giant salamander *Andrias davidianus*, by light microscopy, immunohistochemistry and transmission electron microscopy (TEM) methods. Toluidine blue staining showed TCs with one to two very thin and long telopodes (Tps) that were located in gastric lamina propria. Tps had characteristic structures, including podoms, podomers and dichotomous branching. Immunohistochemistry showed the existence of CD34^+^/PDGFRα^+^ TCs with moniliform Tps in stroma and were close to gastric glands and blood vessels. TEM micrographs also demonstrated the presence of TCs in interstitium between gastric glands. TCs/Tps were located in close proximity to gastric glands, blood vessels, endocrine cells and stem cells. In particular, Tps frequently surrounded stem cells. TCs and Tps, Tps and stem cells established close contacts. Moreover, the exosomes were also found near TCs/Tps. Our data confirmed the presence of TCs in gastric lamina propria of the amphibian, and suggested that TCs cooperate with resident stem cells to regulate endocrine cells and gastric glands regeneration and homeostasis.

Telocytes (TCs) are a novel interstitial/stromal cell reported by Popescu’s team in 2010^1^,^2^,^3^,^4^. A distinctive feature of TCs is presence of one to three very thin and long cell body prolongations with alternating podoms and podomers, called telopodes (Tps)^1^,^2^. TCs can construct a complex 3D network by their long Tps in the interstitial space^5^. Furthermore, TCs can release exosomes to regulate various surrounding cells functions^6^. TCs are suggested to play roles in tissue support, intercellular signaling, cell differentiation, immune surveillance and response, stem/progenitor cells guiding and nursing, homeostasis maintenance, and cell expansion and movement^1^,^4^,^7^,^8^,^9^,^10^,^11^. TCs also participate in tissue hyperplasia, chronic inflammation, organs fibrosis in pathology^12^,^13^. Especially, TCs are suggested to play a key role in tissue regeneration^1^,^2^,^3^,^4^,^9^,^14^. Thus, TCs are believed important for regenerative medicine^15^. Currently, TCs have been demonstrated in many organs of various mammals besides human^16^,^17^,^18^,^19^,^20^. However, knowledge regarding lower animals TCs remains rare. Only a few TCs investigations are performed in several lower animals, such as newt, zebrafish, turtle and chicken^2^,^21^,^22^,^23^. Much less is known about the TCs in amphibians than in any other mammals. Moreover, in contrast to the limited regenerative capacity in the organs of human and mammal, amphibian organs possess stronger regenerative capacity^24^. Therefore, to study regeneration mechanisms, lower animals, for example amphibian, may be a better model animal than mammal. Our previous study has confirmed the presence of TCs in the ileum of an amphibian, Chinese giant salamander *Andrias davidianus* (Amphibia: Caudata)^25^. It is suggested that TCs also may exist in other organs of the amphibian. In order to further investigate whether TCs are also present in other organs of *A. davidianus* and better understand TCs functions, in this study, we employ light microscopy, immunohistochemistry and transmission electron microscopy (TEM) methods, and attempt to identify TCs in the gastric lamina propria of *A. davidianus*.

## Results

### Light microscopy

**Figure 1:**
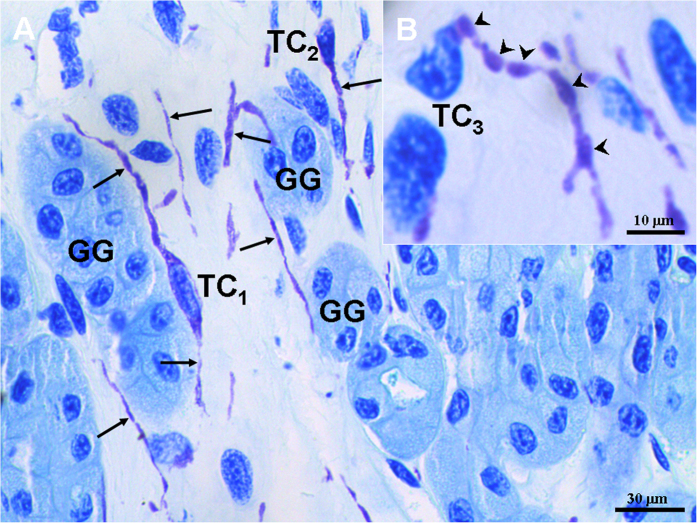
Light microscopy photomicrographs of the gastric lamina propria of *A. davidianus*. The gastric lamina propria histology of *A. davidianus* by toluidine blue staining. (**A**) Telocytes (TC) with long telopodes (Tp) (black arrows) are observed between gastric glands (GG). (**B**) A TC has a long Tp with podoms (black arrowheads), podomers and dichotomous branching.

By toluidine blue staining, TCs with very thin and long Tps were observed in the interstitium between gastric glands (Fig. 1). The cell bodies of TCs contained a big nucleus and a small amount of cytoplasm, and exhibited spindle-shaped and pyriform. The Tp with alternating regions of clear podoms, podomers and dichotomous branching was also observed (Fig. 1B).

### Immunohistochemistry (IHC)

**Figure 2:**
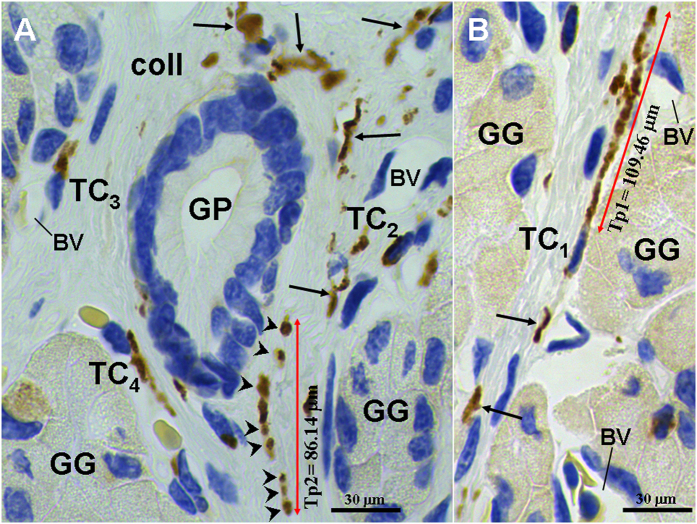
CD34 immunohistochemistry photomicrographs of the gastric lamina propria. (**A**) CD34^**+**^telocytes (TC)/telopodes (Tp) (black arrows) are observed around gastric pit (GP). A long Tp contains clear podoms (black arrowheads) and podomers. (**B**) CD34^**+**^TCs with a very long Tp in the interstitium between gastric glands (GG). BV: blood vessel.

**Figure 3:**
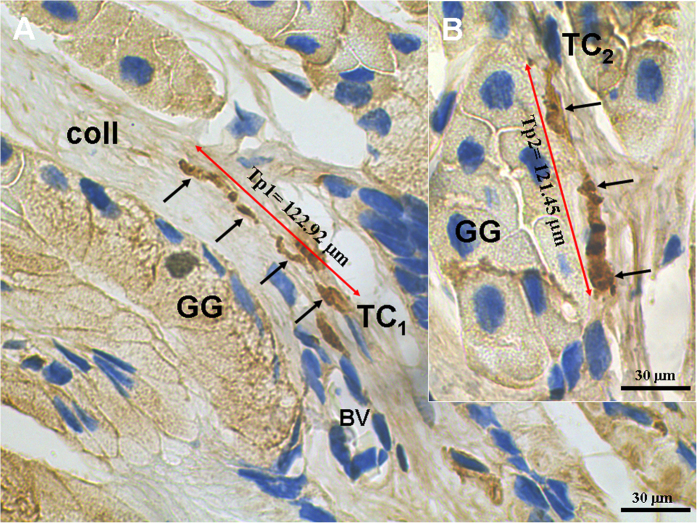
PDGFRα immunohistochemistry photomicrographs of the gastric lamina propria. (**A**) A PDGFRα^**+**^telocytes (TC) with long telopodes (Tp) (black arrows) in interstitium between gastric glands (GG). (**B**) A PDGFRα^**+**^TC close to GG. BV: blood vessel.

CD34^+^/PDGFRα^+^ TCs were located in interstitium between gastric glands (Figs 2 and 3). The cell bodies of CD34^+^/PDGFRα^+^ TCs exhibited spindle-shaped. A characteristic feature of CD34^+^/PDGFRα^+^ TCs was the presence of one to two very long Tps. The average length of all Tps measured on IHC images was 99.20 ± 16.95 μm. CD34^+^/PDGFRα^+^ TCs/Tps were frequently observed in close proximity to gastric glands and blood vessels (Figs 2B and 3B). There were CD34^+^ TCs/Tps in the connective tissue around a gastric pit (Fig. 2A). CD34^+^ Tps presented moniliform because of alternative distribution of podoms and podomers (Fig. 2A). Moreover, the podoms showed stronger CD34^+^.

### Transmission electron microscopy (TEM)

**Figure 4:**
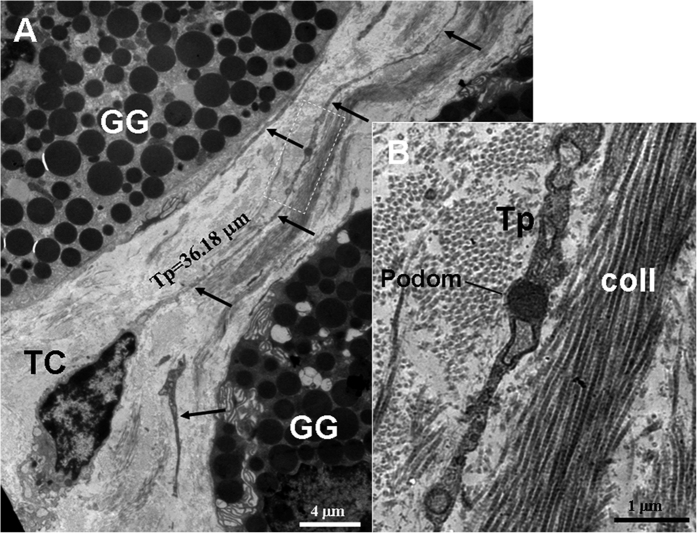
TEM micrographs of the gastric lamina propria of *A. davidianus*. (**A**) A telocyte (TC) with a pyriform cell body and a very long telopode (Tp) (black arrows) is observed between gastric glands (GG) contained many round electron-dense secretory granules. (**B**) High magnification TEM micrograph of the white dashed line boxed area shown in (**A**) with details of a Tp. The podom of Tp can be clearly observed. coll: collagen fibers.

**Figure 5:**
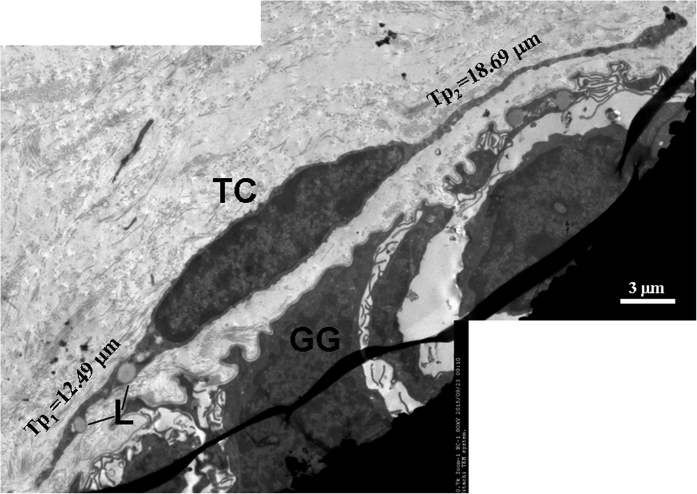
TEM micrographs of the gastric lamina propria of *A. davidianus*. A telocyte (TC) with two long telopodes (Tp) close to gastric glands (GG). L: lipid droplet.

**Figure 6:**
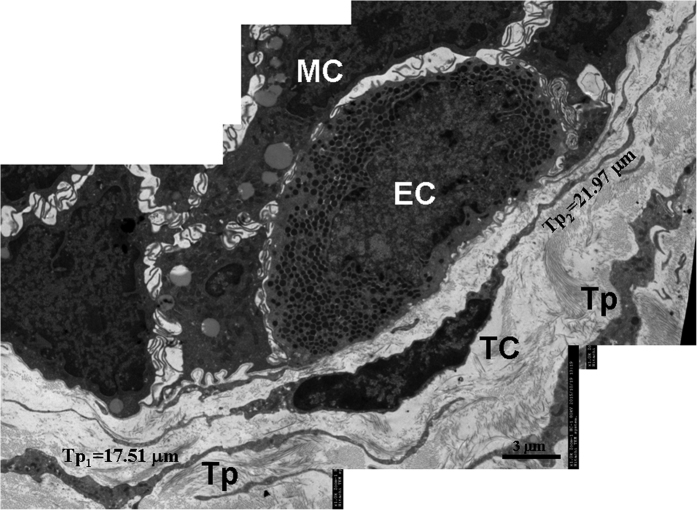
TEM micrographs of the gastric lamina propria of *A. davidianus*. A telocyte (TC) with two thin and long telopodes (Tp) is observed close to an endocrine cell (EC). MC: mucous cell.

**Figure 7:**
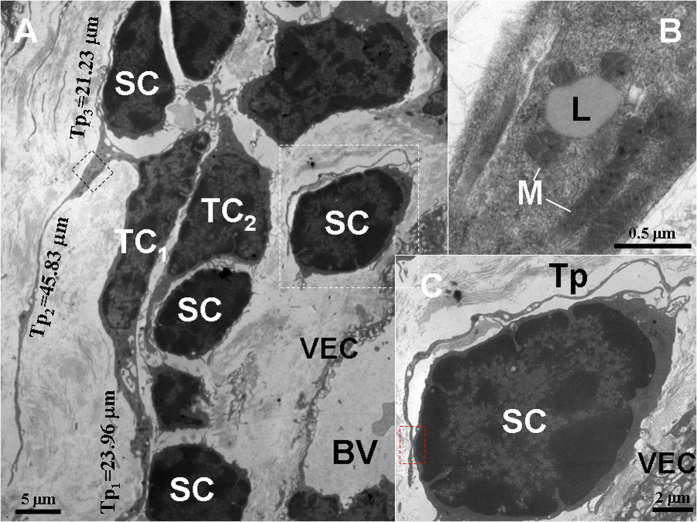
TEM micrographs of the gastric lamina propria of *A. davidianus*. (**A**) Stem cells (SC) and telocytes (TC) with long telopodes (Tp) around a blood vessel (BV). (**B**) The details of a Tp of the black dashed line boxed area shown in (**A**). A lipid droplet (L) and mitochondria (M) in the Tp. (**C**) The details of the Tp and SC of the white dashed line boxed area shown in (**A**). The Tp surrounds the SC, and Tp and SC establishs close contact (red dashed line boxed area). VEC: vascular endothelial cell.

**Figure 8:**
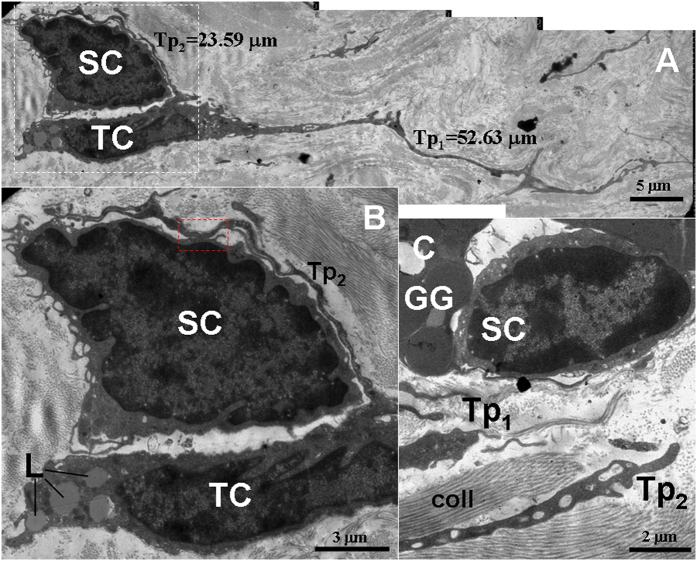
TEM micrographs of the gastric lamina propria of *A. davidianus*. (**A**) A telocyte (TC) with two telopodes (Tp) (Tp_1_ and Tp_2_) is observed. A stem cell (SC) is encompassed with the TC cell body and its Tp_2_. (**B**) High magnification TEM micrograph of the white dashed line boxed area shown in (**A**) with details of cell body and Tp of the TC. The red dashed line boxed area shows the close contact between SC and Tp_2_. L: lipid droplet. (**C**) A SC is observed between gastric glands (GG) and Tp. coll: collagen fibers.

**Figure 9:**
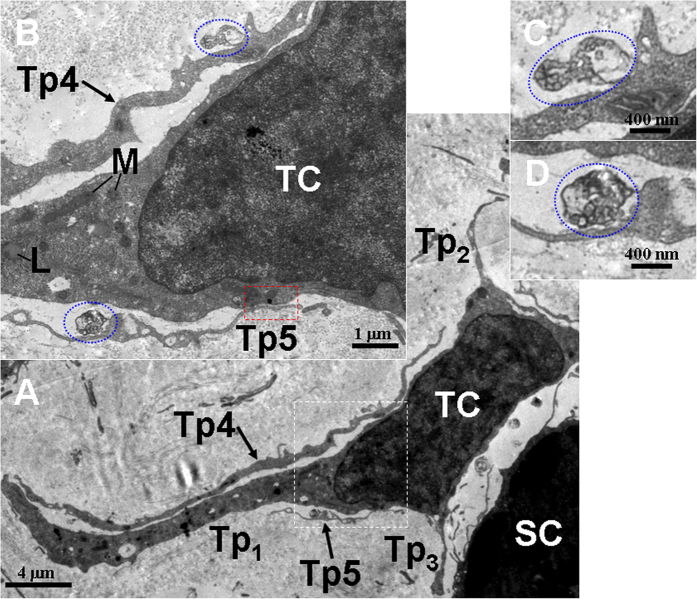
TEM micrographs of the gastric lamina propria of *A. davidianus*. (**A**) A telocyte (TC) with three telopodes (Tp_1_, Tp_2_ and Tp_3_) is observed. In addition, two Tp segments (Tp4 and Tp5) are observed in close proximity to the TC. (**B**) High magnification TEM micrograph of the white dashed line boxed area shown in (**A**). Two Tp (Tp4 and Tp5) contain mitochondria (M). TC with lipid droplet (L) and mitochondria (M) are also observed. The Tp5 and the TC establish close contact (red dashed line boxed area). Two exosomes (blue outlines) are found. The upper exosome is observed adjacent to a Tp. The lower exosome is located between the TC and the Tp5. (**C,D**) High magnification TEM micrographs of the upper and lower blue outline area shown in (**B**) with details of exosomes, respectively. SC: stem cell.

TCs and their long Tps were observed in the connective tissue between gastric glands, which contained many electron-dense and round secretory granules (Figs 4, 5, 6, 7, 8 and 9). The cell bodies of TCs appeared as pyriform (Fig. 4A), spindle-shaped (Figs 5, 6, 7A and 8) and quadrangle (Fig. 9A). The average length of Tps was 27.41 ± 13.10 μm by measured in the TEM images. Some Tps were found in close proximity to gastric glands (Figs 4 and 5). Significantly, a TC with two long Tps was observed adjacent to an endocrine cell, which contained many electron-dense, small and round secretory granules (Fig. 6). Additionally, TCs were frequently observed in close proximity to stem cells (Figs 7, 8 and 9). TCs and stem cells were observed around blood vessel (Fig. 7). Some TCs/Tps closely surrounded stem cells. Moreover, Tps and stem cell established heterocellular close contacts (Figs 7C and 8B). TCs and Tps established homocellular close contacts (Fig. 9B). The exosomes were found between TCs and Tps (Fig. 9B–D).

## Discussion

Previous studies have demonstrated the presence of TCs in the human stomach^26^,^27^. However, in other animals, including lower animals, gastric TCs were not identified. Furthermore, previous TCs identification in human stomach only employed immunohistochemistry and immunofluorescence methods, but the most key diagnostic technique for TCs–TEM was not performed. The ultrastructural illustrations of gastric TCs remain absent regardless of higher and lower animals. In the present study, TCs had extremely long and thin cell processes, i.e. Tps, which appeared as moniliform in morphology due to their characteristic structures, podoms and podomeres. Also, Tps had dichotomous branching. Moreover, TCs/Tps frequently established structure contacts with other TCs/Tps and surrounding cell types. Although fibroblasts and ICC had also cell processes, they were short, thick and cone shaped, and lacked other structures of TCs^3^,^4^. In addition, TCs were CD34^+^ and PDGFRα^+^, while fibroblasts and ICC were PDGFRα^−^ and CD34^− ^26,28. Therefore, we can confirm the existence of TCs in the gastric lamina propria of the lower animal, *A. davidianus*, according to their immunophenotypes and ultrastructural characteristics.

A previous study revealed that the presence of TCs in the mucosa, submucosa and muscle coat of human stomach^26^. Moreover, TCs encircled funds of gastric glands, muscle bundles, blood vessels and nerve structures. The study suggested that TCs might correspond to the fibroblast-like cells. In the other study, Manetti *et al.*^27^ documented the existence of TCs mainly in the muscle layers and myenteric plexus, few TCs in the muscularis mucosae and submucosa of human systemic sclerosis gastric wall. They proposed that TCs loss might have important pathophysiological implications in systemic sclerosis of human organs. These results suggested TCs play physiological and pathological roles in the human stomach. In the present study, TCs/Tps were widely located in interstitium of gastric lamina propria of *A. davidianus*. Furthermore, they frequently were observed in close proximity to gastric gland and blood vessels as well as the reports of Vannucchi *et al.*^26^. Exclusively, by TEM methods, we observed the TCs location frequently close to stem cells. Moreover, we unexpectedly found the TCs/Tps adjacent to endocrine cells. All results suggested TCs and these cell types may be correlative in physiological functions in the gastric lamina propria.

Previous studies also demonstrated that TCs were located in the lamina propria of several animal organs, such as rat duodenum and jejunum, and *A. davidianus* ileum^4^,^11^,^25^. These investigations suggested that TCs may be involved in immune response, intercellular signaling, tissue homeostasis maintenance, and glandular cells secretion and renewal^2^,^4^,^11^,^25^,^29^. Generally, TCs are suggested to control growth and differentiation of other cell types, mainly stem/progenitor cells, and further regulate surrounding cells regeneration and tissue repair in a particular niche^1^,^9^,^30^. In pathological state, they can initiate immune response and trigger tissue inflammation to induce pathogenesis under some challenges^31^. A previous study confirmed that several highly expressed molecules, such as IL-6, VEGF, MIP-1α, MIP-2 and MCP-1, were present in the mouse/rat cardiac TC secretory profile and proteome^32^. These data suggested that the TCs secretome plays a modulatory role in stem cell proliferation and differentiation. In the present study, the TCs were observed around the basal portion of the gastric glands and endocrine cells, where epithelial stem cells are located^33^. Moreover, TCs/Tps were really observed in close proximity to stem cells and they established heterocellular close contacts. Therefore, TCs might be act as nurse cells for stem cells and further to regulate surrounding gastric glands and endocrine cells generation and renewal^1^,^26^. Additionally, TCs might be involved in the glands/hormones synthesis and release of gastric glands/endocrine cells and maintain homeostasis. The homocellular close contacts between TCs and Tps were also present. It is suggested that TCs and Tps can form a 3D network and play a mechanical support role in gastric lamina propria. Furthermore, Tps and stem cells may directly perform intercellular communication by heterocellular close contacts. Additionally, the exosomes were also found near TCs/Tps and stem cells in this study. TCs/Tps and stem cells may also indirectly execute intercellular communication by exosomes^34^, which transfer macromolecules, such as mRNA, microRNAs, proteins and lipids, and participate in obsolete proteins eradication, mediate antigen presentation, stimulate T lymphocyte proliferation, and modulate immune responses^35^,^36^. Accordingly, TCs may utilize exosomes to play various roles^4^,^37^. Whichever communication methods, TCs may communicate with stem cells, nurse resident stem cells, and cooperate with them to regulate gastric glands repair and regeneration^3^,^4^, as well as in another digestive gland–liver^38^,^39^. In addition, TCs and stem cells frequently establish structural connection. The close contact of TCs established with stem cells and the exosomes containing paracrine factors released from TCs might be necessary for stem cells to survive and play roles. It is also suggested that TCs co-culture and co-transplantation with stem cells may more effective and reliable than mono-cellular therapies in regenerative medicine^40^.

## Conclusion

This is the first report to confirm the presence of TCs in an amphibian stomach. Especially, we identified gastric TCs by TEM and disclosed the ultrastructural relation of TCs and other resident cell types, such as gastric gland cells, stem cells and endocrine cells. These results suggest that TCs might play a role in regulating other cell types regeneration and homeostasis. Our data will improve the insight of TCs functions in amphibian stomach.

## Methods

### Animals

All procedures with the *A. davidianus* were conducted by the Ethical Committee for Animal Care and Use of Jiangxi Agricultural University after relevant ethical review according to the National Institutes of Health Guide for the Care and Use of Laboratory Animals. The 2.5-year-old, free range, commercial second filial *A. davidianus* (2 males and 2 females; weight: 0.99–1.12 kg) raised with simulated ecological breeding technology in their primary habitat were generously provided by an artificial breeding farm in mountain area, Jiangxi Province, China. The *A. davidianus* were euthanized on the ice, and then the stomachs were quickly excised and fixed by various fixative solutions for different methods, respectively. The sampling procedures and protocol were approved by the College of Animal Science and Technology, Jiangxi Agricultural University. All the measures are to alleviate animal’ s suffering.

### Light microscopy

The light microscopy technique was conducted according to Zhang *et al.*^41^. The stomachs samples of *A. davidianus* were fixed in 10% neutral buffered formalin for 24 h at room temperature, and then the samples were washed with 0.01 M phosphate-buffered saline (PBS) at pH 7.4, dehydrated in series of graded ethanol, and embedded in paraffin. Sections (5 μm) were cut on a microtome (Yidi, Jinhua, China). After deparaffinization, the sections were stained with toluidine blue solution (0.8% toluidine blue + 0.6% KMnO_4_) for 40 s. After washing in distilled water, the stained sections were dehydrated and mounted. Finally, they were examined and photographed by using a BM 2000 light microscopy with ScopeImage 9.0 (H3D) software (Yongxin, Nanjing, China).

### Immunohistochemistry (IHC)

IHC was performed according to the protocol provided by the anti-CD34 and anti-PDGFRα antibody manufacturer (Abcam, Cambridge, England). Briefly, after deparaffination, the sections (5 μm) were incubated in 3% H_2_O_2_/methanol for 15 min. After washing the sections in PBS (0.01 M pH 7.4) for 3 × 5 min, they were pre-treated using heat mediated antigen retrieval with 0.01 M sodium citrate buffer (pH 6.0) for 15 min, and then washed in PBS for 3 × 5 min. All following steps were carried out in a moist chamber. The sections were incubated in normal goat serum at room temperature for 15 min. After discarding the goat serum, the sections were incubated in the anti-CD34 and anti-PDGFRα antibody (Abcam, Cambridge, England) with diluted in PBS (1:200) at 4 °C overnight, respectively. After rinsing in PBS for 3 × 5 min, the sections were incubated with biotinylated anti-rabbit IgG at 37 °C for 30 min. Afterwards, they were detected using an HRP conjugated compact polymer system at 37 °C for 30 min. The 3,3-diaminobenzidin (DAB) was used as the chromogen. The colouration was terminated with PBS when positive cells were clearly visible. The sections were counterstained with haematoxylin and mounted. Finally, the sections were also photographed by using BM 2000 light microscopy.

### Transmission electron microscopy (TEM)

TEM was conducted according to methods of Yang *et al.*^23^ and Zhang *et al.*^25^. Small pieces of stomachs tissues were fixed in 2.5% glutaraldehyde/PBS (pH 7.4, 0.1 M) at 4 °C for 72 h, and then washed three times in 0.01 M PBS (pH 7.4). The samples were post-fixed in 1% OsO_4_ (Polysciences Inc., Warrington, PA, USA) for 1 h, dehydrated in a concentration series of ethanol, infiltrated with propylene oxide–Araldite mixture and embedded in Araldite. The samples blocks were sectioned at 50 nm by using an ultramicrotome (ReichertJung, Wien, Austria), and then the sections were mounted on cooper coated grids. Finally, these sections were stained with 1% uranyl acetate and Reynold’s lead citrate for 20 min. The stained sections were observed and photographed by a high resolution digital camera connected to the TEM (Hitachi H-7650, Tokyo, Japan).

### Measurement and Statistics

The length of Tps was measured in the light microscopical and TEM images by using Image Pro-plus 7.0 software (Media Cybernetics, Rockville, MD, USA), respectively. Data were analyzed by using Excel software (Microsoft, Redmond, USA).

## Additional Information

**How to cite this article**: Zhang, H. *et al.* Telocytes in gastric lamina propria of the Chinese giant salamander, *Andrias davidianus. Sci. Rep.* **6**, 33554; doi: 10.1038/srep33554 (2016).
